# Mega‐analysis methods in ENIGMA: The experience of the generalized anxiety disorder working group

**DOI:** 10.1002/hbm.25096

**Published:** 2020-06-29

**Authors:** André Zugman, Anita Harrewijn, Elise M. Cardinale, Hannah Zwiebel, Gabrielle F. Freitag, Katy E. Werwath, Janna M. Bas‐Hoogendam, Nynke A. Groenewold, Moji Aghajani, Kevin Hilbert, Narcis Cardoner, Daniel Porta‐Casteràs, Savannah Gosnell, Ramiro Salas, Karina S. Blair, James R. Blair, Mira Z. Hammoud, Mohammed Milad, Katie Burkhouse, K. Luan Phan, Heidi K. Schroeder, Jeffrey R. Strawn, Katja Beesdo‐Baum, Sophia I. Thomopoulos, Hans J. Grabe, Sandra Van der Auwera, Katharina Wittfeld, Jared A. Nielsen, Randy Buckner, Jordan W. Smoller, Benson Mwangi, Jair C. Soares, Mon‐Ju Wu, Giovana B. Zunta‐Soares, Andrea P. Jackowski, Pedro M. Pan, Giovanni A. Salum, Michal Assaf, Gretchen J. Diefenbach, Paolo Brambilla, Eleonora Maggioni, David Hofmann, Thomas Straube, Carmen Andreescu, Rachel Berta, Erica Tamburo, Rebecca Price, Gisele G. Manfro, Hugo D. Critchley, Elena Makovac, Matteo Mancini, Frances Meeten, Cristina Ottaviani, Federica Agosta, Elisa Canu, Camilla Cividini, Massimo Filippi, Milutin Kostić, Ana Munjiza, Courtney A. Filippi, Ellen Leibenluft, Bianca A. V. Alberton, Nicholas L. Balderston, Monique Ernst, Christian Grillon, Lilianne R. Mujica‐Parodi, Helena van Nieuwenhuizen, Gregory A. Fonzo, Martin P. Paulus, Murray B. Stein, Raquel E. Gur, Ruben C. Gur, Antonia N. Kaczkurkin, Bart Larsen, Theodore D. Satterthwaite, Jennifer Harper, Michael Myers, Michael T. Perino, Qiongru Yu, Chad M. Sylvester, Dick J. Veltman, Ulrike Lueken, Nic J. A. Van der Wee, Dan J. Stein, Neda Jahanshad, Paul M. Thompson, Daniel S. Pine, Anderson M. Winkler

**Affiliations:** ^1^ National Institute of Mental Health (NIMH), National Institutes of Health (NIH) Bethesda Maryland USA; ^2^ Leiden University Medical Center, Department of Psychiatry Leiden The Netherlands; ^3^ Leiden Institute for Brain and Cognition (LIBC) Leiden The Netherlands; ^4^ Leiden University, Institute of Psychology, Developmental and Educational Psychology Leiden The Netherlands; ^5^ Department of Psychiatry & Neuroscience Institute University of Cape Town Cape Town South Africa; ^6^ Department. of Psychiatry Amsterdam UMC/VUMC Amsterdam The Netherlands; ^7^ GGZ InGeest Department of Research & Innovation Amsterdam The Netherlands; ^8^ Department of Psychology Humboldt‐Universität zu Berlin Berlin Germany; ^9^ Department of Mental Health University Hospital Parc Taulí‐I3PT Barcelona Spain; ^10^ Department of Psychiatry and Forensic Medicine Universitat Autònoma de Barcelona Barcelona Spain; ^11^ Centro de Investigación Biomédica en Red de Salud Mental Carlos III Health Institute Madrid Spain; ^12^ Menninger Department of Psychiatry and Behavioral Sciences Baylor College of Medicine Houston Texas USA; ^13^ Center for Neurobehavioral Research Boys Town National Research Hospital Boys Town Nebraska USA; ^14^ Department of Psychiatry New York University New York New York USA; ^15^ Department of Psychiatry University of Illinois at Chicago Chicago Illinois USA; ^16^ Department of Psychiatry and Behavioral Health The Ohio State University Columbus Ohio USA; ^17^ Department of Psychiatry & Behavioral Neuroscience University of Cincinnati Cincinnati Ohio USA; ^18^ Behavioral Epidemiology Institute of Clinical Psychology and Psychotherapy, Technische Universität Dresden Dresden Germany; ^19^ Imaging Genetics Center, Mark and Mary Stevens Neuroimaging and Informatics Institute, University of Southern California Marina del Rey California USA; ^20^ Department of Psychiatry and Psychotherapy University Medicine Greifswald Greifswald Germany; ^21^ German Center for Neurodegenerative Diseases (DZNE) Site Rostock/Greifswald Greifswald Germany; ^22^ Department of Psychology Harvard University Cambridge Massachusetts USA; ^23^ Center for Brain Science Harvard University Cambridge Massachusetts USA; ^24^ Department of Psychiatry Massachusetts General Hospital Boston Massachusetts USA; ^25^ Center Of Excellence On Mood Disorders, Department of Psychiatry and Behavioral Sciences The University of Texas Health Science Center at Houston Houston Texas USA; ^26^ LiNC, Department of Psychiatry Federal University of São Paulo São Paulo São Paulo Brazil; ^27^ Section on Negative Affect and Social Processes, Hospital de Clínicas de Porto Alegre, Universidade Federal do Rio Grande do Sul Porto Alegre Rio Grande do Sul Brazil; ^28^ Olin Neuropsychiatry Research Center Institute of Living, Hartford Hospital Hartford Connecticut USA; ^29^ Department of Psychiatry Yale School of Medicine New Haven Connecticut USA; ^30^ Anxiety Disorders Center Institute of Living, Hartford Hospital Hartford Connecticut USA; ^31^ Yale School of Medicine New Haven Connecticut USA; ^32^ Department of Neurosciences and Mental Health Fondazione IRCCS Ca' Granda Ospedale Maggiore Policlinico Milan Italy; ^33^ Institute of Medical Psychology and Systems Neuroscience, University of Muenster Muenster Germany; ^34^ Department of Psychiatry University of Pittsburgh Pittsburgh Pennsylvania USA; ^35^ Department of Psychiatry & Psychology University of Pittsburgh Pittsburgh Pennsylvania USA; ^36^ Anxiety Disorder Program Hospital de Clínicas de Porto Alegre Porto Alegre Rio Grande do Sul Brazil; ^37^ Department of Psychiatry Federal University of Rio Grande do Sul Porto Alegre Rio Grande do Sul Brazil; ^38^ Department of Neuroscience Brighton and Sussex Medical School, University of Sussex Brighton UK; ^39^ Centre for Neuroimaging Science Kings College London London UK; ^40^ School of Psychology University of Sussex Brighton UK; ^41^ Department of Psychology Sapienza University of Rome Rome Italy; ^42^ Neuroimaging Research Unit, Institute of Experimental Neurology, Division of Neuroscience IRCCS San Raffaele Scientific Institute Milan Italy; ^43^ Vita‐Salute San Raffaele University Milan Italy; ^44^ Neurology and Neurophysiology Unit IRCCS San Raffaele Scientific Institute Milan Italy; ^45^ Institute of Mental Health, University of Belgrade Belgrade Serbia; ^46^ Department of Psychiatry, School of Medicine University of Belgrade Belgrade Serbia; ^47^ Graduate Program in Electrical and Computer Engineering, Universidade Tecnológica Federal do Paraná Curitiba Puerto Rico Brazil; ^48^ Center for Neuromodulation in Depression and Stress University of Pennsylvania Philadelphia Pennsylvania USA; ^49^ Department of Biomedical Engineering Stony Brook University Stony Brook New York USA; ^50^ Department of Physics Stony Brook University Stony Brook New York USA; ^51^ Department of Psychiatry The University of Texas at Austin Dell Medical School Austin Texas USA; ^52^ Laureate Institute for Brain Research Tulsa Oklahoma USA; ^53^ Department of Psychiatry & Family Medicine and Public Health University of California La Jolla California USA; ^54^ Department of Psychiatry University of Pennsylvania Philadelphia Pennsylvania USA; ^55^ Department of Psychiatry Washington University St. Louis Missouri USA; ^56^ SAMRC Unite on Risk & Resilience in Mental Disorders, Department of Psychiatry & Neuroscience Institute University of Cape Town Cape Town South Africa

**Keywords:** data sharing, generalized anxiety disorder, mega‐analyses, meta‐analyses, neuroimaging

## Abstract

The ENIGMA group on Generalized Anxiety Disorder (ENIGMA‐Anxiety/GAD) is part of a broader effort to investigate anxiety disorders using imaging and genetic data across multiple sites worldwide. The group is actively conducting a mega‐analysis of a large number of brain structural scans. In this process, the group was confronted with many methodological challenges related to study planning and implementation, between‐country transfer of subject‐level data, quality control of a considerable amount of imaging data, and choices related to statistical methods and efficient use of resources. This report summarizes the background information and rationale for the various methodological decisions, as well as the approach taken to implement them. The goal is to document the approach and help guide other research groups working with large brain imaging data sets as they develop their own analytic pipelines for mega‐analyses.

## Graphical abstract

This report summarizes the background information and rationale for the various methodological decisions made by the ENIGMA—GAD group. The aim of this work is to help guide other research groups working with large brain imaging data sets.

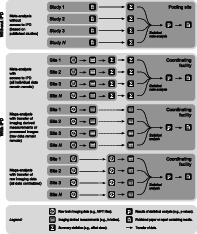

## 1 INTRODUCTION

The ENIGMA (Enhancing NeuroImaging Genetics through Meta Analysis) Consortium, started in 2009, with the aim of performing large‐scale neuroimaging genetics research using meta‐analytic methods by pooling data from around the world. ENIGMA has since expanded to include many working groups, resources, and expertise to answer fundamental questions in neuroscience, psychiatry, neurology, and genetics (Thompson et al., 2014; Thompson et al., 2020). One of these groups is the ENIGMA‐Anxiety working group, created in 2016 (Bas‐Hoogendam et al., 2020), focused on anxiety related disorders. Such disorders, that include social anxiety disorder, specific phobia, panic disorder, generalized anxiety disorder (GAD), and agoraphobia, share substantive phenomenological features and are often comorbid. Within the ENIGMA‐Anxiety working group, a subgroup devoted to the study of GAD was formed, the ENIGMA‐Anxiety/GAD “subgroup,” which for simplicity is referred to here as “ENIGMA‐GAD.”

Because the ENIGMA‐Anxiety working group was formed relatively recently, it has benefited from the experience and work performed by earlier groups, particularly in terms of collaborative methods. In more recent years, research groups have become increasingly favorable toward sharing and transferring de‐identified individual participant data (IPD), often as part of cooperative agreements that respect country‐level differences in data privacy and data protection procedures, discussed below. In the case of ENIGMA‐GAD, as detailed in the final section of this article, the vast majority of sites contributed raw, *T*_1_‐weighted magnetic resonance imaging (MRI) scans, as opposed to processed scans or results of subsequent analyses. These raw data could then be processed centrally using an imaging processing software, in this case FreeSurfer^1^ (Dale, Fischl, & Sereno, 1999; Fischl et al., 2002; Fischl, Sereno, & Dale, 1999). Having access to raw IPD provided unique opportunities to review methods for handling and harmonizing such data, defining processing pipelines, and implementing analytic strategies. Crucially, this led ENIGMA‐GAD to prioritize a mega‐analysis approach. This approach consists of analyzing IPD from all sites in one stage. This contrasts with two‐stage approaches, which consist of analyzes of site‐specific results in a second step after each site generates processed data in an initial step (detailed below).

This paper presents some of the challenges posed by the decision, by the ENIGMA‐GAD group, to use a mega‐analysis, and discusses the rationale for the choices that were made to establish the analysis plan. The discussion is broadly applicable to mega‐analyses in the context of ENIGMA and other international neuroimaging efforts. Below, differences between meta‐analytic vs. mega‐analytic approaches, benefits of preregistration, issues concerning data sharing and data reuse are discussed. Methods for quality control and choices with respect to measurements and statistical analyses are also presented. Finally, specific choices in the ENIGMA‐GAD group with respect to each of these issues are described.

## 2 META‐ANALYSIS VERSUS MEGA‐ANALYSIS

As collaborative and coordinated endeavors, ENIGMA meta‐analyses studies operate differently from literature‐based meta‐analyses. In the latter, results from published studies are compiled to draw conclusions on a certain question. In most cases, such pooled studies have been conducted and published over many years, with high sample and methodological heterogeneity, encompassing diverse statistical approaches. Such diversity is aggravated in meta‐analyses that examine neuroimaging studies. In neuroimaging studies, substantial challenges for combined inference result from the use of statistical maps limited to significant *p*‐values or test statistics, tables with coordinates in relation to some standard (but not always the same) stereotaxic space, and different representations of the brain (volume‐based or surface‐based; Fox, Lancaster, Laird, & Eickhoff, 2014; Müller et al., 2018; Tahmasian et al., 2019). Moreover, because of publication biases, there can be a misrepresentation of negative results (the “file‐drawer” problem; Rosenthal, 1979) or study selection (Roseman et al., 2011).

**FIGURE 1:**
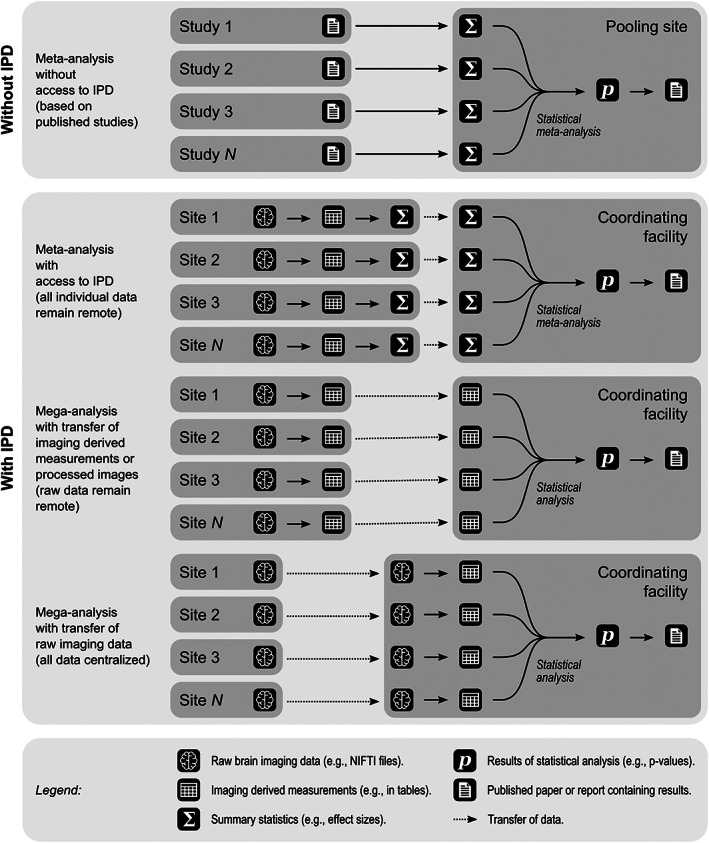
Differences between classical, literature‐based meta‐analyses, conducted without access to individual participant data (IPD) (upper panel) versus approaches used by different ENIGMA working groups, in which researchers, collectively, have access to IPD (lower panel). The latter encompasses three main approaches (top) data are processed using common methods at each site, then summary statistics are computed and sent to a coordinating facility which then conducts a meta‐analysis; (middle) data are processed using common methods at each site, then sent to the coordinating facility which then conducts a mega‐analysis; and (bottom) raw data are sent to the coordinating facility which then processes the data in batch and conducts a mega‐analysis, while taking site‐specific effects into account

In ENIGMA, these issues are minimized through analysis of IPD using an agreed upon processing strategy. Briefly, three approaches, that relate to data location, are currently used by different projects within ENIGMA working groups: (a) all raw data and all derived IPD remote in relation to the coordinating facility; (b) all raw data remote in relation to the coordinating facility, but derived data centralized; (c) all raw data centralized. These approaches are not mutually exclusive within a working group, and different projects conducted by the same working group may each use a different strategy, depending on the project goals and considerations about data availability, computational resources, and expertise. These approaches are summarized schematically in Figure 1.

For meta‐analysis with access to IPD, the strategy includes quality checks and statistical analysis over mostly coetaneous data. Summary statistics (such as effect sizes, standard errors, and/or confidence intervals) are pooled by a coordinating facility that then uses meta‐analytic methods for inference across sites. Such a coordinated, two‐stage meta‐analysis approach has been pursued by most ENIGMA working groups (Hibar et al., 2015; Hibar et al., 2016; Schmaal et al., 2016; Stein et al., 2012; van Erp et al., 2016), particularly due to privacy concerns regarding genetic data. ENIGMA genome‐wide association studies still use a meta‐analysis approach (Hibar et al., 2017; Satizabal et al., 2019); sites analyze their own data with an agreed upon protocol, which avoids the need to transfer individual participant genomic data, and allows distributed analysis of computationally intense approaches.

Other strategies can be considered if the coordinating facility has access to all IPD: a single‐stage statistical analysis can be performed by the coordinating facility, while addressing site‐related heterogeneity; this would be a one stage meta‐analysis, or simply “mega‐analysis.” With imaging data, such mega‐analyses could start with the raw images being sent to the coordinating facility where they then undergo batch processing using identical methods and computing environments. Alternatively, mega‐analyses could start with image‐derived measurements, such as the volumes of brain structures or cortical surface area, already computed and furnished by the participating sites to the coordinating facility, for each individual participant; the coordinating facility then proceeds to the statistical analysis. Combination of approaches for some projects (e.g., some sites sending raw data for processing whereas others sending processed data) are also possible.

Analyses using IPD offer several advantages (Riley, Lambert, & Abo‐Zaid, 2010): they improve consistency in inclusion criteria across sites, better treatment of confounds and of missing data, verification of assumptions of statistical models, standardization of procedures, increases in statistical power, reductions in biases for not depending on previous publications of (invariably significant) results. Access to IPD further allows other strategies for investigation that are not limited to hypotheses testing. For example, it may allow classification at the individual participant level using machine‐learning methods (Nunes et al., 2018). In a mega‐analysis starting with raw imaging data, all data can be processed identically in the same facility, thus minimizing the chance for errors or variability that can arise when each site conducts these aspects of the analysis. One major challenge to this approach is that mega‐analysis requires at least one site to possess the necessary resources and expertise to handle large datasets. Additionally, this approach is only possible when IPD are shared with a central facility. Data exchange on an IPD level often is limited as data protection is regulated differently among research projects, consortia, and countries. Barriers on data exchange and limitation of available resources can, in effect, restrict the participation to few well‐equipped centers.

Multiple studies have compared meta‐ and mega‐analysis (Belias, Rovers, Reitsma, Debray, & IntHout, 2019; Riley et al., 2010; Simmonds, Stewart, & Stewart, 2015), suggesting superiority of mega‐analyses with IPD when compared to meta‐analyses in terms of higher statistical power and acceptable false positive rates. In the context of ENIGMA, comparisons have likewise tended to favor mega‐analyses (Boedhoe et al., 2019; Kochunov et al., 2014; Koshiyama et al., 2020). However, if individual sites use identical processing strategies with IPD, a random‐effects two‐stage approach leads to the same estimates as a (one‐stage) mega‐analysis. This is well‐established in the neuroimaging literature, which uses similar statistical methods for multi‐level inference for analysis of functional magnetic resonance imaging data (Beckmann, Jenkinson, & Smith, 2003; Worsley et al., 2002). Rarely such identical processing can be accomplished, though, given the usually large number of sites and, and the need that all engage in approaches intended to ensure consistency (discussed below).

## 3 ANALYSIS PLAN AND PREREGISTRATION

Preregistration of clinical trials has been emphasized for many years, and a registry^2^ was established by law in the United States through the Food and Drug Administration Modernization Act of 1997 (Dickersin & Rennie, 2003). Similar registries exist in other countries, and an international directory was created by the World Health Organization (WHO), the International Clinical Trials Registry Platform (ICTRP).^3^ However, broadly similar efforts did not emerge in other research areas for decades. Defining a hypothesis, an associated analysis plan, and preregistering these ideas before conducting any analyses is important in many ways (Chambers, 2013). It helps to conceptually separate specific, previously formulated hypotheses from exploratory analyses that have potential to generate new hypotheses based on the data (Nosek, Ebersole, DeHaven, & Mellor, 2018; Wagenmakers, Wetzels, Borsboom, van der Maas, & Kievit, 2012). Likewise, it helps to separate a priori and exploratory hypotheses and the analytic plans used for their investigation (Ledgerwood, 2018). The benefits, however, stretch well beyond epistemological advantages by reducing the potential for questionable research practices (Chambers, Feredoes, Muthukumaraswamy, & Etchells, 2014). For example, preregistration reduces problems that follow when negative results remain unreported (Rosenthal, 1979; Sterling, 1959), reduces the chances of selective reporting (Macleod et al., 2014) and maximizes transparency in analytic approaches, thereby facilitating replication (Simmons, Nelson, & Simonsohn, 2011). Without preregistration, these problems remain prevalent, possibly due to the structure of incentives in academic environments (Neuroskeptic, 2012; Nosek, Spies, & Motyl, 2012). Preregistration also reduces hypothesizing after the results are known (Kerr, 1998), and protects scientists from other biases (Chambers, 2013), such as confirmation bias, hindsight bias, and anchoring effects (Moreau, 2019).

For ENIGMA, specific details and challenges need to be considered when preregistering a study. First, an analytic plan must be discussed with participating centers. The plan should include who access the data, roles of each participating site and their personnel, compliance with supervening laws and regulations, funding sources, as well as authorship expectation. This ensures that pooled data from different cohorts are analyzed in a way acceptable by all investigators. Second, many ENIGMA sites may have already analyzed the data they share for meta or mega‐analysis, often to test similar hypotheses as those being considered for the ENIGMA combined analyses. Obtaining credible results requires an analytic plan free of influences from findings known by the investigators, and that remains inclusive of all relevant data. Preregistration mitigates such concerns by supporting reasonable hypotheses of broad interest and with well‐defined inclusion and exclusion criteria of subjects, both of which are unlikely to be swayed by prior knowledge of outcomes. These analytic plans are formalized into “project proposals,” which can be distributed to members for approval and participation, and are often considered a form of preregistration for working group members.

Many platforms support preregistration, though the platform provided by the Open Science Foundation^4^ stands out for its comprehensiveness and user‐friendliness. The process is remarkably simple, with the site offering detailed instructions and preregistration templates. Specifying an embargo period before the registration becomes public is possible, and a digital object identifier (DOI) can be generated.

## 4 DATA SHARING AND REUSE

Both meta‐ and mega‐analysis require that individual sites transfer data to the coordinating facility. Aggregated data, such as histograms of quality metrics, effect sizes, confidence intervals, and standard errors, are not identifiable at the individual level and can be transferred parsimoniously among sites without substantive risk of reidentification. It should be noted, however, that without precautionary measures, repeated computation of aggregate results using slightly varying subsets of participants can expose information about individuals (Dwork, 2006). This risk can be minimized through agreements among researchers on the nature and amount of aggregated data to be transferred. For mega‐analyses, in which IPD are transferred, further attention is needed, due to differences across sites in the regulations that protect the confidentiality, integrity, and security of the IPD and their use in human research. In international collaborations, such as ENIGMA, accommodating such requirements necessitates that the strictest regulations are followed. While compliance with the law must be integral, three points are particularly relevant for ENIGMA projects: (a) protection of data and privacy of research subjects, (b) data reuse, and (c) international transfers of data.

In the United States (US), research must follow the Federal Policy for the Protection of Human Subjects (the “Common Rule”; Arellano, Dai, Wang, Jiang, & Ohno‐Machado, 2018). This requires that specific consent be obtained from participants before their data and/or specimens can be used not only for the research project for which they are enrolling, but also for future research that may use such material, which often is the case of ENIGMA projects. Privacy in the US is governed by the Health Insurance Portability and Accountability Act (HIPAA) of 1996, which requires patient data to be de‐identified; reuse requires approval by an Institutional Review Board.

Regulations differ, however, across countries. In the US, there is a presumption that processing personal data is lawful unless it is expressly forbidden. In the European Union (EU), in contrast, the processing of such data is prohibited unless there is a lawful basis that permits it (Dove, 2018). Legal provision for data protection and use in research comes from the General Data Protection Regulation (GDPR), adopted in 2016, which also covers the use of data from EU residents outside the Union (Chassang, 2017). While HIPAA emphasizes subject privacy, the GDPR makes no direct mention of privacy whatsoever, dealing instead with data protection, as established in the EU Charter of Fundamental Rights along with the right to a private life. Privacy is extremely difficult to define (Alfino & Mayes, 2003), and may be understood in this context as a state of nonaccess to data pertaining to an individual (Dove, 2018). Data protection, in turn, is a less ambiguous definition and can be understood as a set of rules that aim to protect the rights, freedoms, and interests of individuals whose personal data are handled and used (Tzanou, 2013).

The GDPR establishes that data reuse should only be allowed where new purposes are compatible with those for which the data were initially collected. This is usually the case for ENIGMA analyses. International data transfers are not allowed unless the country to which data are sent has been found by the European Commission to provide “adequate” data protection; at the time of this writing, the list of countries for which an adequacy decision has been provided includes, for example, Argentina, Israel, Japan, New Zealand, and Switzerland.^5^ While the list does include the US and Canada, in the case of these two it does so for commercial uses of data that do not broadly cover research by universities and research institutes as needed for ENIGMA. In the absence of such adequacy decision, or of specific derogations, an alternative path to data transfer is through specific provision of safeguards concerning data protection. These require the signing of legally binding agreements between authorities, or binding corporate or institutional rules approved by competent supervisory authorities (Dove, 2018; Staunton, Slokenberga, & Mascalzoni, 2019). If none of these paths are viable, a possible solution to still allow research is to determine that the coordinating facility for a given ENIGMA Working Group will be in the EU itself; then no data from EU subjects need to be transferred to outside the Union. However, such a workaround is limited in scope and time: countries that are in the process of adopting legislation modeled after GDPR (such as the United Kingdom through the Data Protection Act of 2018) will be under broadly similar rules; these countries might, nonetheless, quickly receive an adequacy decision by the European Commission, such that transfers between the EU and these countries should ultimately be facilitated.

### 4.1 De‐identification

Regardless of specific legislation, data de‐identification is a crucial step. De‐identification consists of removal of personally identifiable information that allows data to be traced back to individuals, thus rendering such identification impossible or extremely difficult or unlikely. In the context of HIPAA, unless otherwise determined by an expert, removal of information such as names, locations with granularity smaller than that of a state, dates related to an individual (such as birth date, admission date, etc.), and other identifying details, is considered to provide a reasonable basis to assume that the information cannot be used to identify an individual. Full‐face photographs and any comparable images must likewise be removed for HIPAA compliance. For ENIGMA data, this means that MRI scans may need to have facial features of subjects removed before data are shared (see below).

Unlike HIPAA, the GDPR does not specify de‐identification methods. Instead, researchers are expected to remain mindful that de‐identified data might become reidentifiable through the development of new technologies or use of ancillary data. Thus, the GDPR requires vigilance to ensure that data remain anonymous (Dove, 2018). Managing the risk of reidentification is crucial, and safeguards should be put in place as if the data were not anonymous. Pseudonymized (e.g., tokenized or key‐coded) data are subject to the GDPR, even if the codes are not shared and remain within different organizations. For ENIGMA, this means that sites that handle information of EU residents must ensure complete de‐identification as well as take into account the risk that de‐identified data becomes reidentifiable, or pursue GDPR compliance by treating data as if not anonymous.

Imaging data stored in the standard Digital Imaging and Communications in Medicine (DICOM) file format are accompanied by a host of personally identifiable information. Tools exist to anonymize such files, by erasing fields from the file header that could contain such information. Another popular file format used in brain imaging is the Neuroimaging Informatics Technology Initiative (NIFTI). This format stores no personally identifiable information but contains two general‐purpose fields (“descrip” and “intent_name,” with 80 and 16 bytes, respectively) that could hold such information. The format can also accommodate extensions, and can be paired with a JavaScript Object Notation text file (JSON), both of which may contain information that may allow subject identification. Any field with information that could lead to reidentification must be erased or removed before data can be shared between ENIGMA sites and the coordinating facility, or other safeguards must be in place to ensure no reidentification will be attempted or possible. A popular tool for conversion from DICOM to NIFTI, “dcm2niix” (Rorden, 2014) allows removal of such information during format conversion.

Moreover, the data portion of DICOM and NIFTI files may be edited to ensure that facial features will be removed (defacing). Reidentification of participants based on scan data had been considered a remote possibility, which motivated the creation of defacing algorithms (Alfaro‐Almagro et al., 2018; Bischoff‐Grethe et al., 2007; Milchenko & Marcus, 2013; Schimke, Kuehler, & Hale, 2011). Such reidentification, however, has recently been demonstrated to be feasible (Schwarz et al., 2019), which now renders defacing mandatory for publicly available data. Moreover, two recent developments further complicate matters. First, even defaced data may be reidentified, particularly if facial features are only blurred, as opposed to completely removed (Abramian & Eklund, 2019). Second, recent research indicates that defacing unfortunately may degrade the performance of image processing algorithms, possibly affecting the quality of measurements obtained (de Sitter et al., 2020). For ENIGMA mega‐analyses, reconciling data protection with maximum scientific value that data can provide may ultimately require bilateral agreements to avoid data breaches that could allow for unintended or malicious use. In this case the participating institutions can reach an understanding (usually in the terms of a data use agreement—DUA) that all shared data is to remain securely stored with limited access to researchers who are conducting relevant ENIGMA work.

### 4.2 Encryption and transfer

Encryption reduces the possibility that data might be misappropriated when stored, or intercepted during transfer, and thus reduces the chances that data can be used in ways that are not in the best interest of research participants. Data encryption is always compatible with both HIPAA and GDPR, and in the case of the former, it can be considered “a reasonable and appropriate measure” to ensure confidentiality, which renders it mandatory for all practical purposes. Even without specific regulations, data encryption is good practice insofar as the confidentiality, integrity, and security of data of participants are concerned.

A basic scheme consists of encrypting the data using a reasonably secure cipher (algorithm), with a key (password) that can also be used for decrypting. Such a key is transmitted from an individual site to the ENIGMA coordinating facility through means other than those used to transfer the encrypted data. A more sophisticated approach uses pairs of public/private keys: the site encrypts the data using the public (not secret) key provided by the coordinating facility; data can be decrypted by the coordinating facility using the private (secret) key. Various tools enable encryption of individual files or the generation of encrypted containers, which are files that emulate a file system and can hold multiple other files. Examples of such tools that operate across multiple platforms include VeraCrypt,^6^ CipherShed,^7^ and GnuPG.^8^

Data transfer can be performed in different ways: if data are strongly encrypted, transmission does not require further encryption; if data are not encrypted, transmission should use a form of secure communication. For small amounts of data, such as for analyses using imaging‐derived measurements, which tend to be smaller than with imaging data in full resolution, a plain email with encrypted attachments containing the (possibly compressed) data, or encrypted emails with nonencrypted attachments, are sufficient. For large volumes of data, as for mega‐analyses that start from raw data, data transfer using methods such as SSH file transfer protocol (SFTP) are more convenient. However, connection to institutional servers (or even personal laptops) hosting the data may require the potentially problematic opening of firewall exceptions. Two alternative methods are straightforward to implement. One uses strong ciphers to encrypt the data, which are then stored in a physical portable medium, such as external hard drives, thumbsticks, or even secure digital (SD) cards, to be sent by post or courier; decryption keys are negotiated ahead of time and shared through different means. The second method uses peer‐to‐peer secure transfers using a service such as Globus,^9^ a nonprofit, free service provided by the University of Chicago (Ananthakrishnan, Chard, Foster, & Tuecke, 2015) for data exchange among academic or research institutions.

Cloud storage systems (e.g., Dropbox,^10^ Box,^11^ Amazon Web Services,^12^ Google Drive,^13^ and Microsoft Azure^14^) should be used with caution. Even though most cloud providers offer some level of encryption, compliance with data protection and privacy laws may only be offered with high tier subscriptions or specific security settings, if offered at all. Users should be aware of the level of compliance that their choice of cloud system provides.

Encryption and transfer have sometimes to be established on a site‐specific basis. Some sites may have particular expertise and/or infrastructure in place to allow transfer of large amounts of data using particular methods, which would be favored over others. Laws governing transfer of technology and geopolitics may also impact choices: furnishing encryption software to some countries is illegal in some jurisdictions, whereas receiving hard drives may also pose difficulties in countries that heavily tax or delay the delivery of imported goods.

### 4.3 Organization and processing

Before or after being transferred to the coordinating facility, the data can be organized into a scheme that facilitates processing and the use of imaging pipelines, such as the brain imaging data structure (BIDS; Gorgolewski et al., 2016). BIDS prescribes a hierarchy of files and directories that is simple and intuitive, yet powerful enough to accommodate a diverse set of imaging modalities collected in varied circumstances. The scheme is intended to minimize efforts related to data curation, to reduce the number of errors due to incorrect organization of data files, and to facilitate the development and usage of software, which can be written to parse the file structure directly (Gorgolewski, Alfaro‐Almagro, Auer, Bellec, & Capot, 2017).

Processing of the whole dataset using one operating system and software version can help avoid inconsistencies. It has been demonstrated that differences in operating systems can have a small effect on, for example, FreeSurfer metrics (Gronenschild et al., 2012); such metrics have been used in many ENIGMA analyses to date, including in ENIGMA‐GAD analyses. Scientists may benefit from monitoring their computing environment and run analyses in batches that are not interspersed with periodic software updates.

Options to ensure software consistency include the use of virtual machines (such as QEMU/KVM,^15^ VirtualBox,^16^ or VMware^17^) or containerized environments (such as Docker^18^ or Singularity^19^). In virtual machines, the whole system—including emulated hardware and the “guest” operating system—can be kept static and be shared. Containers use a layer of compatibility between the “host” operating system and the desired applications. They tend to run faster and have simpler maintenance than virtual machines. In either case, the researcher can keep tight control over software versions, libraries, and dependencies. Neither of the two methods, however, is ideal. Virtual machines can be heavier to run and offer less flexible integration with the host operating system (which in turn may have access to a large computing cluster, such that integration is something often desirable). Containers address this problem but introduce others: troubleshooting experimental software may be difficult because it is not always clear whether a given problem has arisen because of the software itself, or because of the container or its interaction with the host system. Regardless, such solutions improve reproducibility of results by allowing researchers to share not only their code and information about their computing environment, but also their actual computing environment.

## 5 QUALITY CONTROL

For ENIGMA meta‐analyses, each site can perform a quality assessment of its own data using a previously agreed protocol. Sites can report the quality metrics to the coordinating facility, which then can use the information in the statistical model by, for example, giving less weight to sites contributing lower‐quality data. ENIGMA protocols provide consistent, streamlined strategies for visual inspection of imaging data; these strategies involve inspection of the cortical border between gray and white matter, parcellations of the cortex, and segmentation of subcortical structures. For mega‐analyses, while the same kind of visual inspection could be advantageous, the amount of data may render this process difficult. Although there is no standard triage or similar requirement before sharing raw data, it is usually the case that images will have already been seen by at least one investigator before sharing, and as such, might have been excluded from consideration and not sent to the coordinating facility. Moreover, while using the same raters may give higher consistency on selection of participants across sites given imaging features, the same process might introduce unwanted bias toward selection, for example, if imaging features used to visually define inclusion or exclusion are unknowingly related to the variables investigated, a risk that may be present even if quality criteria are consistent across sites.

### 5.1 Automated methods

Biases arising from manual inspection can be minimized through automated quality control methods. In the UK Biobank, for example, a supervised learning classifier identifies problematic images with acceptable accuracy (Alfaro‐Almagro et al., 2018). The UK Biobank, however, benefits from the fact that data collection is limited to only three sites, all of which use identical equipment (Miller et al., 2016). In ENIGMA, data come from many sites, with MRI scanners from different vendors and models, with different field and gradient strengths, different coils, acquisition sequences, and software versions. Using a quality control classifier with such heterogeneous data is challenging (Chen et al., 2014; Focke et al., 2011; Han et al., 2006; Jovicich et al., 2006), although methods with good performance have been proposed (Klapwijk, van de Kamp, van der Meulen, Peters, & Wierenga, 2019).

**FIGURE 2:**
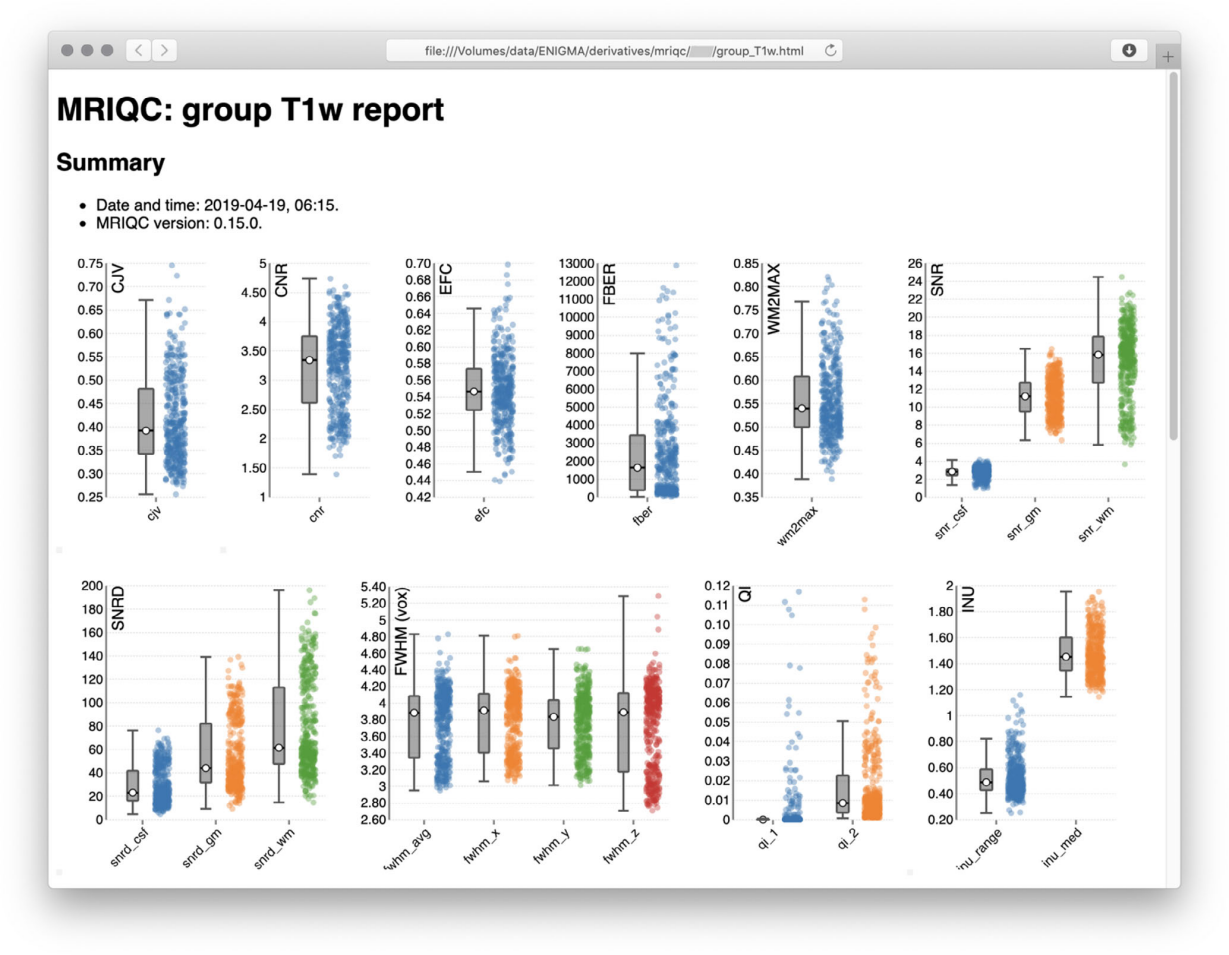
Example screenshot of a report of image quality for the subjects of one site. Box plots of various metrics are shown. The report is produced by the tool MRIQC, available, along with documentation that details all the metrics (many more than shown in the figure), at https://mriqc.readthedocs.io

Tools such PCP‐QAP^20^ (Zarrar et al., 2015) and MRIQC^21^ (Esteban et al., 2017) compute a host of image quality metrics that consider signal, noise, image smoothness and contrast, as well as specific artifacts (Atkinson, Hill, Stoyle, Summers, & Keevil, 1997; Dietrich, Raya, Reeder, Reiser, & Schoenberg, 2007; Ganzetti, Wenderoth, & Mantini, 2016; Magnotta & Friedman, 2006; Mortamet et al., 2009). In particular, MRIQC operates on data organized according to BIDS, and produces detailed reports of these metrics. This tool does not, however, classify images as having high or low quality; instead, it provides an interface for a rater to make that determination based on the computed quality metrics and possibly other features; these metrics may, in turn, be used to train a classifier. Such classification, however, can be difficult to generalize, given the diversity of data from multiple sites (Esteban et al., 2017). Even so, derived metrics may be insufficient to predict successful generation of cortical surfaces and segmentation of subcortical structures with FreeSurfer, from which image‐derived measurements of interest are often computed. Notwithstanding these considerations, it is good practice to investigate quality using this kind of tool, which includes boxplots (Figure 2), and mosaics that show multiple slices color‐coded so as to highlight potential defects. The output from these tools are useful to assist in flagging images that, even if successful at FreeSurfer processing, may require specific decisions whether or not they should remain in the sample. Moreover, these tools provide summary metrics that can be returned to the contributing sites, where local researchers can assess the quality of their own images versus those collected by others or elsewhere (Esteban et al., 2019).

### 5.2 Euler characteristic

One particular metric has been found to be a good predictor of the quality of FreeSurfer outputs: the Euler characteristic (*χ*; sometimes also called Euler number) of the cortical surface produced before topological correction (Rosen et al., 2018). To conceptualize the Euler characteristic, consider a polyhedron whose spatial configuration is determined by its vertices, edges, and faces. It can be shown (Lakatos, 1976) that if the polyhedron is convex, the number of vertices (*V*), minus the number of edges (*E*), plus the number of faces (*F*), is always equal to 2; this quantity is the Euler characteristic, that is, *χ* = *V* − *E* + *F*. If the polyhedron is crossed by a single hole, *χ* is decreased by 1; if crossed by two holes, decreased by 2; if hollow, *χ* is increased by 1. More generally, for every hole that crosses a polyhedron, its *χ* is decreased by one, whereas for every hollow, it is increased by one. The Euler characteristic is well‐known in neuroimaging as a key metric for multiple‐testing correction using the random field theory (RFT; Worsley et al., 1996). Here, however, it serves an entirely different purpose: it acts as a metric to quantify topological deviation of the initial cortical surface from a sphere, as an increasingly large number of holes in the initial surface generates an increasingly negative Euler characteristic. As these values become more negative, the more likely it is that the original *T*_1_‐weighted scans had low quality in ways that negatively impacts the surface reconstruction. FreeSurfer treats such holes as topological defects and corrects them automatically to create a cortical surface that reaches a *χ* = 2 (Fischl, Liu, & Dale, 2001). However, initial surfaces that have too many defects are less likely to be ever usable, even after topology correction.

The Euler characteristic was found to be highly correlated with manual quality ratings, discriminating accurately unusable from usable scans, and outperforming other data quality measures (Klapwijk et al., 2019). However, the precise threshold to be applied to *χ* remains unknown when deciding whether a surface is usable or not; such a threshold may be site or scanner specific. Moreover, it is not currently known whether, as a general rule, the Euler characteristics for each brain hemisphere should be combined as their mean, or the worst (minimum, most negative) of the two, nor whether other metrics related to surface topology could be helpful for quality assessment. For subcortical structures, specific quality metrics are currently missing from the literature.

### 5.3 Manual edits

Image processing pipelines may allow manual edits when automated approaches fail to generate processed images of desirable quality. This is also the case with FreeSurfer, whereby the user can employ “control points” to establish final cortical surface placements; segmentation also can be hand edited to exclude nonbrain tissue and fix mislabeled regions. Different ENIGMA working groups have decided differently on whether or not to do manual editing, as the process is time consuming and requires expert knowledge. Crucially, while manual editing may improve validity of measurements, it introduces variance to the data unrelated to the images themselves, but related to the manual operator; multiple operators potentially compound the problem. Taken to an extreme, such undesired variance can reduce power, though research has found that such editing may have little impact on the final results, in either a beneficial or deleterious manner (McCarthy et al., 2015).

## 6 MEASUREMENTS

Imaging generates a myriad of measurements. Analyses can reveal genetic and environmental influences on healthy and pathological variability in the human brain, providing great potential currently not fully harnessed. As an example, a recent ENIGMA meta‐analysis using data from 51,665 subjects identified 187 loci influencing cortical surface area and 12 others influencing thickness (Grasby et al., 2020); in another example, the recent UK Biobank analysis used 3,144 imaging‐derived traits (Elliott et al., 2018) to find 148 replicable clusters of associations between single nucleotide polymorphisms and these traits. Not all imaging traits are sufficiently well defined and stable to allow reliable quantification for ENIGMA meta or mega‐analyses, though. While the problem can be partially mitigated in mega‐analyses that use common processing schemes, stable, reliable measurements should be the first line of research. Region‐based or vertex‐based measures of cortical thickness, cortical surface area, and cortical and subcortical volumes are easily obtained, and measurement workflows are established across multiple research sites (Hibar et al., 2015; Stein et al., 2012; Thompson et al., 2014). Diffusion‐weighted imaging also allows measurements that are robust to variations on processing pipelines, and workflows for ENIGMA have been developed (Jahanshad et al., 2013; Kochunov et al., 2015). Likewise, a resting‐state functional MRI processing pipeline was proposed recently for use in ENIGMA meta‐analyses (Adhikari et al., 2018).

The goal of these pipelines is to ensure consistency in methods across sites, not affecting the relationship between the imaging measurements and their underlying biological processes. In effect, such measurements may be influenced by a myriad of physiological and pathological processes. For cortical thickness and surface area, for example, measurements depend to some extent on neuronal and glial cell volume and density, dendritic complexity, degree of myelination, inflammatory processes, and other factors. Evidence has been reported for changes in cortical thickness associated with learning (Zatorre, Fields, & Johansen‐Berg, 2012) and even with stimuli during structural MRI data collection (Månsson et al., 2020); the latter finding, if confirmed, could indicate that information about what the subjects viewed would need to be considered as a confound at the time of the statistical analysis.

Furthermore, the scale with which measurements are obtained does not necessarily correspond to the scale in which linear effects manifest; such linear effects constitute the backbone of most statistical brain‐imaging analyses as encapsulated in the general linear model (GLM). The GLM assumes that modeled factors (e.g., diagnostic group, age, and sex) possess additive effects over the dependent variable (e.g., an imaging‐derived measurement); this may not always hold. Some notable examples include fine‐resolution area of the cortex, which follows a lognormal distribution potentially reflecting exponential influences; fractional anisotropy of water diffusion, a quantity bounded between 0 and 1, also could be considered not a sum of multiple small effects, nor functional connectivity assessments bounded between −1 and 1. Cases such as these may be accommodated through the use of a data transformation, such as logarithmic, power, Fisher's *r*‐to‐*z*, logit, or probit transformations; generalized linear models and nonparametric statistics can also be considered.

### 6.1 Choice of resolution

Researchers need to consider whether imaging analyses should use measures obtained at every point of an image (e.g., voxelwise or vertexwise data) or aggregate measures computed over regions of interest or parcellations, broadly termed as “ROIs.” Although vertexwise analyses have been performed in recent ENIGMA research (Chye et al., in press; Ho et al., 2020), most previous ENIGMA studies used meta‐analyses. In these cases, an ROI‐based approach is more robust to small deviations from a common image registration scheme. Moreover, voxelwise and vertexwise measurements represent small pieces of tissue in relation to the resolution inherent to the equipment or scanning sequence. As such, these measures are intrinsically noisier than ROI‐based quantities. Furthermore, because the number of voxels/vertices is usually many times larger than the number of ROIs under potential consideration, their use is computationally more intensive, and leads to an exacerbation of the multiple testing problem.

These considerations, however, do not imply superiority of ROI‐based measurements over voxelwise or vertexwise approaches. While noisier, vertexwise and voxelwise data are typically smoothed, thereby increasing the signal‐to‐noise ratio while retaining localizing power. Moreover, statistical power of ROI‐based measurements is maximal when the space spanned by the true effects matches perfectly the borders of the ROI; otherwise, true signal is diluted within an ROI, or split across multiple ROIs. Multiplicity of testing, while more severe with voxelwise or vertexwise data, may not necessarily compromise power: the tests are largely nonindependent and methods to accommodate such nonindependence exist, both in parametric (Worsley et al., 1996) and nonparametric cases (Winkler, Webster, et al., 2016). Finally, it is not always obvious how to aggregate measurements for ROIs, nor what the ROIs should be. For example, while the surface area of an ROI can be trivially obtained by summing together the areas assigned to all vertices within that region, thickness within the same ROI could be computed either as an average of all vertices, or as a weighted average using the areas of the vertices as weighting factor; for functional MRI, aggregate measurements could be the simple average, or the first principal component. Moreover, a host of different parcellation schemes exist (Craddock, James, Holtzheimer, Hu, & Mayberg, 2012; Desikan et al., 2006; Destrieux, Fischl, Dale, & Halgren, 2010; Glasser et al., 2016; Ji et al., 2019; Maggioni, Tana, Arrigoni, Zucca, & Bianchi, 2014; Power et al., 2011; Schaefer et al., 2018; Tzourio‐Mazoyer et al., 2002; Yeo et al., 2011), based on various macroscopic, microscopic, and functional aspects of the brain, none of which is clearly superior to others for all possible investigations (Arslan et al., 2018; Brett, Johnsrude, & Owen, 2002; Messé, 2019). For instance, the use of an anatomical parcellation in fMRI studies might hide possibly relevant functional inhomogeneities within each cluster (Maggioni et al., 2014).

**FIGURE 3:**
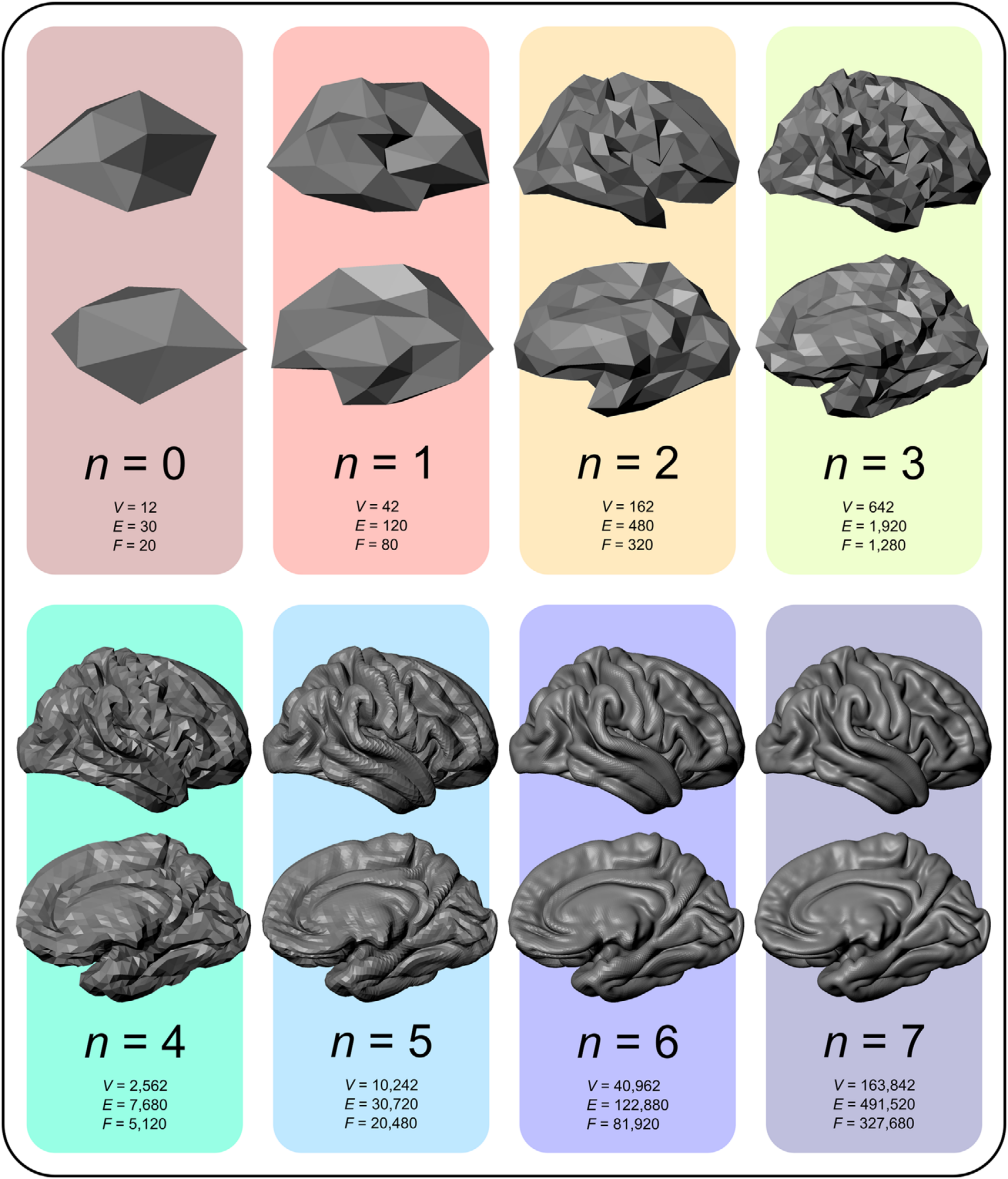
Surface reconstructions of the cortex of the right hemisphere based on different resolutions of a recursively subdivided icosahedron. The default in FreeSurfer uses *n* = 7 recursions, resulting in a total of 163,842 vertices. Considerable computational savings can be obtained with lower resolutions (such as with *n* = 4 or 5) without substantial losses in localizing power. *V*, number of vertices; *E*, number of edges; *F*, number of triangular faces

Pooling raw IPD for mega‐analysis creates many data analytic opportunities. Since the coordinating facility has access to all data, mass‐univariate analyses are possible without the constraints imposed by the limited data exchanges of meta‐analyses. Mass‐univariate methods support easier, more reliable forms of vertexwise/voxelwise analyses, performed by processing all data in an identical manner, regardless of site provenance, an approach that has already been used in ENIGMA (Wang et al., 2019). However, this process still can be computationally difficult. FreeSurfer default surface data, for example, uses 163,842 vertices per hemisphere for between‐subject comparisons, which is denser than the number of voxels that pass through the pia mater or the interface between gray and white matter in a typical MRI scan, given the cortical convolutions; moreover, data are usually smoothed, further lowering the effective resolution. Computational savings that do not substantively sacrifice localizing power may be accomplished by downsampling the surface. In the case of FreeSurfer, this downsampling can be done by using an icosahedron recursively subdivided fewer times (*n*) than the default 7 (Winkler et al., 2012, appendix A) as the target for interpolation. Data from vertices not explicitly included are still represented when smoothing is applied before interpolation, thus before downsampling. The number of vertices is given by *V* = 10 · 4^*n*^ + 2 if the grid is based on an icosahedron; using *n* = 4 or 5 still allows good cortical coverage (Figure 3).

### 6.2 Harmonization

In a mega‐analysis, pooling data from numerous cohorts requires addressing nuisance factors. Site, scanner and cohort‐specific effects of no interest can be manifest as effects larger than diagnosis or other effects of interest; neglecting such nuisance effects can reduce power or generate false positives and low reproducibility (Baggerly, Coombes, & Neeley, 2008; Leek et al., 2010). These confounds can be accommodated at the time of the statistical modeling and analysis, at the penalty of increasing model complexity, or the data may be modified before analysis so as to remove such unwanted effects.

ComBat (“combining batches”) is such an approach, that allows harmonization of data across sites. The method originated in genetics for correcting batch effects in microarrays, and is described in detail in (Johnson, Li, & Rabinovic, 2007). In brief, ComBat incorporates systematic biases common across voxels/vertices, under the mild assumption that phenomena resulting in such “batch” effects (e.g., site, scanner, and/or cohort effects) affect voxels/vertices in similar ways (e.g., stronger mean values, higher variability). In the method, location (additive) and scale (multiplicative) model parameters that represent these batch effects are estimated. This estimation is done by pooling information across voxels or vertices from participants from each site so as to shrink such unwanted effects toward an overall group effect (i.e., across batches and voxels/vertices). These estimates are then used to adjust the data, robustly discounting unwanted effects. Variability of interest or related to known nuisance or confounds (e.g., age or sex) can be retained. In brain imaging, the approach has been an effective method to harmonize diffusion tensor imaging data (Fortin et al., 2017), cortical thickness measures (Fortin et al., 2018), rest and task‐based functional MRI (Nielson et al., 2018), and functional connectivity (Yu et al., 2018). ComBat has been used in ENIGMA studies (Hatton et al., in press; Villalón‐Reina et al., in press), although it has been argued that it leads to similar results as random effects linear regression (Zavaliangos‐Petropulu et al., 2019). Which statistical harmonization model is optimal remains an active discussion at the time of this writing.

## 7 STATISTICAL ANALYSIS

Statistical analyses can proceed once data have been processed, and measurements obtained and possibly harmonized. Such analyses estimate the effects of interest and compare them to expected observations should there be no real effect to compute a p‐value. It is at the stage of the statistical analysis that the differences between meta‐ and mega‐analysis become most pronounced.

### 7.1 Fixed versus random effects

For all cases discussed in Figure 1, analyses may assume that true effects are fixed (constant) across sites, and therefore any differences in effects among sites are solely due to random experimental error, or may assume that the true effects themselves may be random (i.e., varying) across sites. For meta‐analyses without access to IPD, the above distinction between fixed and random effects holds relatively without ambiguity, and distinct methods to summarize literature findings for either of the two cases exist (Borenstein, Hedges, Higgins, & Rothstein, 2009). For other cases, unfortunately, these terms have multiple meanings that sometimes conflict (Gelman, 2005). For research using IPD, less ambiguous definitions apply to slopes and intercepts, which can be treated as constant (thus, fixed) or allowed to vary (thus, random) across sites. This distinction between fixed and random becomes then an attribute not of the statistical model, but of each independent variable.

As for the level of inference, in the case of ENIGMA, the selection of sites is seldom a random quantity, and generalization is sought not to an idealized “population of sites”, but instead to the actual population. Thus, between‐site variability is a nuisance that should either be modeled by including random intercepts to accommodate different site means, or be addressed through data harmonization, as discussed above. Effects of interest, such as differences between individuals with a specific condition and comparison individuals, can be assumed to be fixed across sites (thus, would be modeled as a single regressor, i.e., with fixed slopes), or assumed to vary across sites (thus, would be modeled with multiple regressors, i.e., with random slopes), thus implying the possibility of an interaction of site by effect of interest. The latter would accommodate, for example, site differences due to clinical characteristics or recruitment setting. Unwanted effects other than the intercept can be modeled either considering fixed or random slopes, the consideration being left on a per variable basis. For example, age effects may be modeled using fixed slopes if all sites have participants within similar age ranges, but using random slopes if some sites have only young participants whereas others have only elderly, as age is often not expected to have a linear effect across lifespan.

### 7.2 Confounds

Unwanted data variability may arise due to procedural factors including site or scanner features, or due to factors that affect both dependent and independent variables. Variables representing the former case are termed *nuisance*; those representing the latter, *confounds*. Variables such as age or sex may be nuisance in some analyses or confounds in others, depending on the relationship between these variables and the other variables studied; here we broadly call nuisance and confound variables *covariates*.

The large sample size of ENIGMA increases statistical power in general, however, this may result in erroneous labeling confounding effects (Smith & Nichols, 2018); ignoring such confounds may reduce power or identify spurious associations. Addressing these concerns can be challenging, as decisions regarding confounding variables affect interpretation of the relationship between dependent and independent variables (Gordon, 1968; Lord, 1967). For example, if a confounding variable causes at least part of the variation observed in the imaging data across participants and in the variation of the independent variable (i.e., it is a *collider*), adjustment for the undesired effect induces a false association (Berkson, 1946; Luque‐Fernandez et al., 2019; Pearl, 2009, chapter 6), which can happen in either direction (positive or negative).

Moreover, controlling for poorly reliable measures may not completely remove their putative effects, leading to false conclusions about effects (J. Westfall & Yarkoni, 2016). While this is a greater concern for social and psychological constructs that often are measured with relatively low reliability, the same can apply to imaging measurements. Examples include segmentation of structures where tissue contrast is minimal, or for structures that are small for the image resolution; for functional MRI, false conclusions can occur through effects of signal fluctuations that are poorly associated with task performance or of weak functional connectivity among regions. All such measures can produce variables that, if used as confounds, may increase the chance of false positives. Perhaps counterintuitively, here too large sample sizes may exacerbate the problem.

A special kind of confound in brain imaging is a composite measurement formed by pooling together values of all voxels/vertices or regions of interest, with the goal of discounting unwanted global effects. For example, in a vertexwise analysis of surface area, it might be of interest to consider the total cortical surface area as a confounding variable. Likewise, for studies of subcortical volume, total brain size—or a related quantity, the intracranial volume (Buckner et al., 2004)—can be considered a confound; for cortical thickness, the average thickness across the cortex; for functional MRI, at the subject level, a measurement of global signal, though controversial, might be considered in a similar manner (Murphy & Fox, 2017). The rationale for inclusion of a global measurement as a regressor within the model stems from interest in enhancing the localizing power afforded by imaging methods, and reducing sources of noise that affect measures globally. From this perspective, the scientist seeks to learn where, specifically, in the brain some phenomenon may occur. In this context, arguably, global effects would be of lesser interest, unless a research hypothesis is specifically about them. In addition, for functional MRI, some sources of noise, such as movement and respiration, result in artifactual global signal changes, and so removal of the global signal is also an effective means of reducing artifacts (Ciric et al., 2017).

What makes these confounds special is that, being composites of all other local (voxelwise/vertexwise) or regional quantities, they are almost certainly correlated with these measurements, and thus, are likely to also be associated with variables of interest in the model if these are associated with the local or regional measurements. These global variables are more likely to impact results where local or regional effects of interest are present, even more so if these are widespread across the brain. Options for taking into account such global effects in the statistical analysis have been studied (Andersson, 1997; Barnes et al., 2010; Nordenskjöld et al., 2013; Sanfilipo, Benedict, Zivadinov, & Bakshi, 2004). The main approaches are: (a) convert each local or regional measurement into a proportion over the global quantity; (b) residualize the dependent variable with respect to the global; and (c) include the global in the model. Among these three, the latter option should always be favored as it accounts for effects that the confounding variable may have over both dependent and independent variables. The least preferable is the proportion method (a), one of the reasons being that noisier (unreliable) measurements compromise the measurements to a much greater extent than the others.

If confounding variables are meant to be included in the model, it is often appropriate, considering all the above, to present results with and without these variables in the model (Hyatt et al., 2020; Simmons et al., 2011). Ideally, these would also be corrected for multiple testing, as the number of opportunities for falsely significant results has now doubled (see below more on multiple testing).

### 7.3 Inference

Choices for inference can be broadly divided into parametric and nonparametric. Parametric methods are computationally faster but require assumptions that are sometimes difficult to justify. For example, data have to be assumed to be independent and normally distributed with identical variances after all nuisance variables and confounds have been taken into account. These assumptions may hold for some analyses, but not for others. When the variety of imaging modalities possible for ENIGMA studies is considered, these assumptions cannot hold for all of them. The consequence is that results will be incorrect in at least some cases. Nonparametric tests, such as permutation tests, on the other hand, require very few assumptions about the data probability distribution, and therefore can be applied to a wider variety of situations than parametric tests. For permutation tests, the only key assumption is that any random instantiation of permuted data must be as likely to have been observed as the original, unpermuted. In other words, the data must be *exchangeable*. If exchangeability holds, permutation tests are exact, in the sense that the probability of observing a *p*‐value smaller than a predefined significance level *α* is *α* itself when there are no true effects (Holmes, Blair, Watson, & Ford, 1996; T. E. Nichols & Holmes, 2002; Winkler, Ridgway, Webster, Smith, & Nichols, 2014).

For ENIGMA mega‐analyses, permutation tests can pose practical challenges, though. Large sample sizes and the multiplicity of sites, combined with modeling that include random slopes and random intercepts for covariates, leads to large design matrices that can be slow to process repeatedly as needed for permutations. Moreover, unless data have been harmonized in ways that accommodate potential different variances across sites, statistics that are robust to heteroscedasticity (DiCiccio & Romano, 2017; Guillaume, Hua, Thompson, Waldorp, & Nichols, 2014; Winkler et al., 2014) can likewise add to the computational burden; here, permutations may be restricted to blocks of exchangeable observations that have been collected within each site or within scanner. Another increase in computational expense occurs if more powerful, yet nonstandard test statistics, such as pseudo‐*t* (T. E. Nichols & Holmes, 2002), or spatial statistics such as cluster extent, cluster mass, or threshold‐free cluster enhancement (TFCE) (Smith & Nichols, 2009) are used. In all these cases, speed can be increased using fast, parallel implementation of permutation algorithms (Eklund, Dufort, Villani, & Laconte, 2014), or using accelerations based on various mathematical and statistical properties of these same tests (Winkler, Ridgway, et al., 2016), or both.

### 7.4 Multiple testing

As with any imaging experiment that uses one statistical test per imaging element (voxel, vertex, ROI), correction for multiple testing is necessary (T. Nichols & Hayasaka, 2003). For parametric inference, and under a series of additional assumptions, it is possible to control the familywise error rate (FWER) using the RFT (Worsley et al., 1996); methods and software exist for both voxelwise and vertexwise data. However, this method cannot be used for ROIs, as these cannot be represented as a regular lattice, or for voxelwise data that do not meet all the assumptions of the theory, such as tract‐based spatial statistics (Smith et al., 2006). A valid approach for all these cases, but that controls a different error quantity, is the false discovery rate (FDR) (Benjamini & Hochberg, 1995; Genovese, Lazar, & Nichols, 2002). For permutation inference, correction for multiple testing that controls the FWER can be accomplished in a straightforward manner for all the above cases using the distribution of the maximum statistic obtained across all tests in each permutation (P. H. Westfall & Young, 1993). Uncorrected permutation p‐values can also be subjected to FDR correction.

Correction should consider not only the multiplicity of imaging elements as voxels or ROIs, but also the multiple imaging‐derived measurements eventually tested in the same ENIGMA study (e.g., cortical thickness and cortical area), as well as multiple hypotheses formulated in terms of contrasts of parameters of the model (Alberton, Nichols, Gamba, & Winkler, 2019), or multiple models for the same data, for example, with and without a global measurement as confounding variable. Failing to consider these issues exposes a study to the risk of excess false positives. Correcting across multiple tests for multiple hypotheses in the same study is challenging with parametric tests given the existing but invariably unknown dependence structure among tests; Bonferroni correction, while valid, is unduly conservative given that dependence. Correction using the distribution of the maximum statistic, assessed via permutations, solves the problem, regardless of the dependence structure, yielding exact results, thus that are neither conservative (thus not less powerful given the multiplicity of tests) nor invalid.

## 8 REPORTING RESULTS

Classical meta‐analyses results are often reported with the aid of forest plots (Borenstein et al., 2009), which show effect sizes and confidence intervals for each study separately (or for each site in the case of ENIGMA), along with a combined effect size that considers the effects from all studies after some sensible weighting. ENIGMA studies that used meta‐analyses adopted a similar approach where possible, for example, when imaging metrics were collapsible into single numbers, such as asymmetry (Guadalupe et al., 2017; Kong et al., 2018) or indices for specific structures (Hibar et al., 2015; Stein et al., 2012). For mega‐analyses, while such plots may be of lesser value as the ultimate conclusions come from pooling all IPD into a single analysis, reporting forest plots may still be helpful for showing potentially distinct effects at each site, as well as identifying outlier sites and qualitatively revealing how disperse the data are. However, for this purpose, the mega‐analysis may need to be broken down into separate analyses, one per site, or contrasts tested separately for each site in the case of random slopes (which accommodate interactions of effects of interest by site). While these two approaches are equivalent if there are no fixed slopes or fixed intercept anywhere in the model, running analyses separately for each site is computationally less intensive (and can be done in parallel in a straightforward manner). Voxelwise and vertexwise results cannot, however, be feasibly shown with forest plots, and the usual, color‐coded maps for effect sizes and/or *p*‐values in logarithmic scale become then necessary, one per site, as well as for the overall results.

### 8.1 Authorship

Given the large number of involved sites and investigators, authorship of published reports are an important aspect of ENIGMA projects. While there are no enforceable rules to determine the authorship of a scientific paper, a number of organizations have provided guidelines and recommendations intended to ensure that substantial contributors are credited as authors; for a review, see (Claxton, 2005). One such organization is the International Committee of Medical Journal Editors (ICMJE, also known as the “Vancouver group”), which recommends that authorship are based on the following four criteria: (a) substantial contributions to the conception or design of the work; or the acquisition, analysis, or interpretation of data for the work; (b) drafting the work or revising it critically for important intellectual content; (c) final approval of the version to be published; (d) agreement to be accountable for all aspects of the work in ensuring that questions related to the accuracy or integrity of any part of the work are appropriately investigated and resolved. It is recommended that all four conditions are satisfied. Moreover, all authors should be able to identify which co‐authors are responsible for other specific parts of the work (ICMJE, 2019).

Most ENIGMA studies have tried as much as possible to adhere to these recommendations. For example, while not all investigators from all sites may contribute directly to the planning or execution of the meta‐ or mega‐analysis, intellectual formulation of research hypotheses and study design that led to the data collection at a given site often are the same for which the data or results were pooled across multiple ENIGMA sites, which, together with data collection itself, satisfies the first criterion. Collaborative, real‐time text editing tools, such as Google Docs,^22^ Authorea,^23^ and Overleaf,^24^ allow many authors to work simultaneously on the same document, editing and providing each other with comments, thus satisfying the second and third criterion. The fourth recommended criterion may be satisfied implicitly through the communication established during the editing process, by assenting upon the publication of a preprint, at a time in which an author may choose to opt out before submission to a journal and where eventual rectifications are more complex, or by signing a form in which sugh agreement is made explicit. ENIGMA working group members who do not satisfy the Vancouver criteria may be presented with alternatives, such as (a) be named under a consortium author if they worked on overarching conception of the project but not on the specific paper, (b) be named in the Acknowledgments section of the paper, or (c) not named; in the absence of guidelines, participating members are free to choose what best represents their contribution.

## 9 MEGA‐ANALYSIS IN THE ENIGMA‐GAD GROUP

Having discussed the above, we are now in a position to better describe the specifics of the ENIGMA‐GAD analyses. In this group, sites were contacted based on their publication and funding record using imaging data of subjects with a history of anxiety disorders, and who could meet criteria for GAD. Virtually all sites that were contacted and that did have structural imaging data were able to participate. While imaging offers a large range of measurements, the group began by examining structural, *T*_1_‐weighted imaging scans and performing an analysis based on FreeSurfer. These choices reflected the popularity of these scans, which are nearly universally collected, regardless of other imaging modalities that each site may have used for their investigations. Furthermore, FreeSurfer‐based pipelines and quality control protocols were already available from previous ENIGMA studies. Having these as a starting point facilitated the establishment of a new working group.

As described above, IPD were made available by the participating sites to the coordinating facility—in this case, the National Institute of Mental Health (NIMH), part of the US National Institutes of Health (NIH)—such that a mega‐analysis could be conducted. While the superiority of mega‐analyses is established, another factor was the proportion of sites contributing IPD: nearly all sites preferred to send raw, anonymized imaging scans, as opposed to individual level processed data or simply summary results. Another early decision involved preregistering the analytic plan, which was deposited at the Open Science Foundation, where it remains publicly accessible.^25^ The plan was registered after data had been received by the NIMH and processed using FreeSurfer, such that sample sizes were known (eventually more sites could contribute data after overcoming institutional barriers; these were included in the analysis), and before statistical analyses. Data from some sites may have been part of previous publications; the team analyzing the data at the coordinating facility did not deliberately check the existence of previous results, nor whether previous analyses on subsets used similar or different methods. Instead, inclusion and exclusion criteria for data were based on diagnoses given to the subjects in relation to the research hypotheses, and the ability of the sites to make their data available to the coordinating facility. In the preregistered plan, research hypotheses were specified, inclusion and exclusion criteria were defined, dependent and independent variables were indicated, and statistical methods outlined. Exploratory analyses that were not the main focus of the study were also listed.

A de‐identification agreement was signed between the NIMH and each participating center whereby any eventual identifiable data would not be disclosed or requested by any of the parties. De‐identified data were transferred using the Globus service to a storage partition of the high performance computing (HPC) systems of the NIH. Each site had its own endpoint for transfer, such that no information of any kind could leak from one contributing site to another.

**FIGURE 4:**
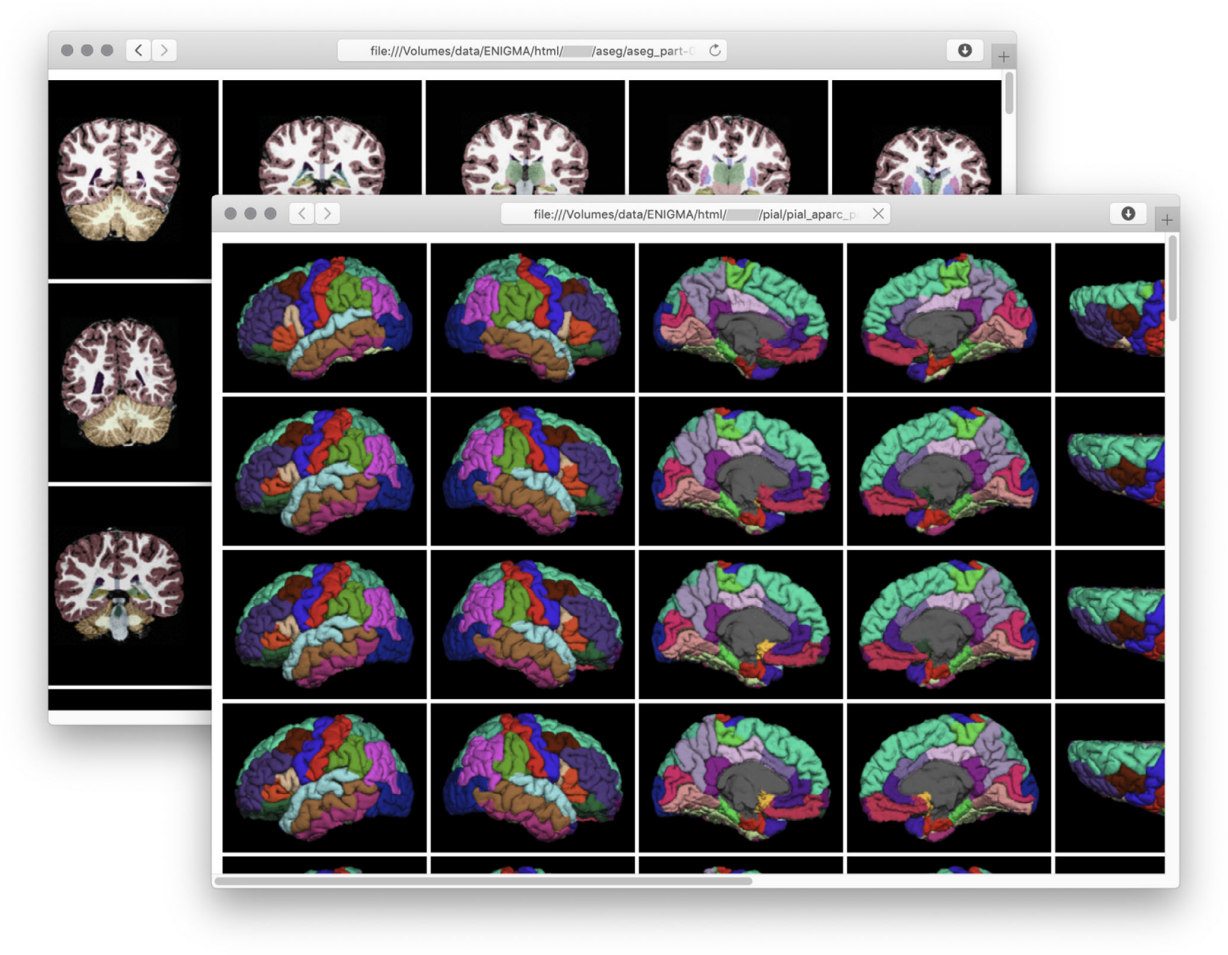
Example report pages with multiple views of the cortical surfaces (front) and slices of subcortical volumes (back). Pial surfaces are shown, but inspection can use white and inflated; slices with subcortical volumes can be complemented with surface overlays. The script that generates these pages uses FreeSurfer scripting to automate the operation of the tools “tkmedit” and “tksurfer,” and is available at https://brainder.org

The received data were organized according to BIDS, passed through an initial quality check using MRIQC, and processed using the FreeSurfer 6.0.0. Given the large number of subjects, it was not viable to follow the ENIGMA‐QC^26^ protocol, according to which every cortical parcel and every subcortical region of every participant would need to be visually inspected and annotated where usable or not, so as to give a mark “pass” or “fail.” This process would require an excess of person‐hours not available to the group. Instead, a semi‐automated method was used. First, a script^27^ to quickly allow visual inspection of FreeSurfer cortical surfaces and subcortical segmentations of many subjects in a single report page was used (Figure 4). One of the benefits is that, by showing many subjects at the same time, the page permits one to quickly learn how good quality surface and segmentations should look like. Reconstructions with clear defects that grossly affected anatomy were marked for exclusion by a single researcher for all the data; all other reconstructions were, at this stage, not yet marked for exclusion. Second, from the results of FreeSurfer, the Euler characteristic of the surfaces before topology was obtained (it is stored in the log file “recon‐all.log”), as well as the number of vertices of these surfaces. Receiver operating characteristic curves relating variation on the Euler characteristic threshold and the ability to reject the surfaces marked for exclusion after visual inspection were constructed so as to identify site‐specific thresholds. Ultimately, the Euler characteristic was replaced by the ratio between the Euler characteristic and the number of vertices in the surfaces before topology correction, as this measure had a better ability to discriminate between good and bad surfaces in this dataset. Finally, the identified threshold was used to determine which subjects would be included and which would not, on a per site basis.

Measurements considered for analyses, as indicated in the preregistration and as in previous ENIGMA studies, included cortical measurements of thickness and surface area for each of the parcels of the Desikan–Killiany atlas (Desikan et al., 2006), as well as volumes of subcortical structures. Cortical vertexwise thickness and surface area were also measured, and downsampled to the resolution of an icosahedron recursively subdivided four times, with 2,562 vertices per hemisphere. Because sites differed widely in variables such as age, modeling age with random slopes (with additional quadratic effects) seemed more appropriate than merely assuming that, across all ages and sites, age effects would be exactly the same. Models with and without a global measure (total surface area, average thickness, and intracranial volume) were considered. Correction for multiple testing used the distribution of the maximum statistic, assessed via permutations. ComBat was not used for the main analyses; instead, scanner‐specific effects were modeled (random intercepts) and a test statistic robust to heteroscedasticity was used, along with variance groups (one per site) and exchangeability blocks. ComBat is, however, being assessed with the same data as a potential option for future studies; results will be reported opportunely. Statistical analysis for this mega‐analysis used the tool Permutation Analysis of Linear Models (PALM).^28^ At the time of this writing, the analysis is being finished and the manuscript is being prepared for publication (Harrewijn et al., in prep.). Authorship, like with the present paper, was defined according to Vancouver criteria, generally with early career investigators, members of the coordinating facility and who worked directly with the data handling and the bulk of the writing appearing first, and with lead investigators appearing last; in between, the contributing sites in alphabetic order, and, within each site, early investigators appearing first; lead investigators last.

## 10 CONCLUSION

This overview described the analytic choices across the various stages of an ENIGMA mega‐analysis, setting out the reasoning behind these choices. Aspects related to data protection and privacy, and how to handle confounds, along with other challenges that inevitably occur when large‐scale data from multiple sites are analyzed were also discussed. The various choices made by ENIGMA‐GAD when facing each of the discussed topics were presented. The hope is that the resulting survey of these practical considerations will be useful to others embarking on similar multi‐site neuroimaging studies, especially those integrating data across multiple countries and data modalities.

## DISCLOSURES

Paul M. Thompson received partial grant support from Biogen, Inc., (Boston, MA) for research unrelated to this manuscript. Anderson M. Winkler received in the past support from the Government of Brazil through the Conselho Nacional de Desenvolvimento Científico e Tecnológico (CNPq: 211534/2013–7). Straube was funded by the Deutsche Forschungsgemeinschaft (DFG, German Research Foundation), Projects C07/C08, SFB‐TRR 58. Dan J. Stein has received research grants and/or honoraria from Lundbeck and Sun. Hans J. Grabe has received travel grants and speakers honoraria from Fresenius Medical Care, Neuraxpharm, Servier, and Janssen Cilag as well as research funding from Fresenius Medical Care.

## Data Availability

Data sharing is not applicable to this article as no new data were created or analyzed in this study.

## ENDNOTES

^1^

http://freesurfer.net

^2^

https://www.clinicaltrials.gov

^3^

https://www.who.int/ictrp

^4^

https://osf.io

^5^
The complete and updated list can be found at https://ec.europa.eu.

^6^

https://www.veracrypt.fr

^7^

https://www.ciphershed.org

^8^

https://www.gnupg.org

^9^

https://www.globus.org

^10^

https://dropbox.com

^11^

https://box.com

^12^

https://aws.amazon.com

^13^

https://drive.google.com

^14^

https://azure.microsoft.com

^15^

http://www.linux-kvm.org

^16^

https://www.virtualbox.org

^17^

https://www.vmware.com

^18^

https://www.docker.com

^19^

https://sylabs.io/singularity

^20^

http://preprocessed-connectomes-project.org

^21^

https://mriqc.readthedocs.io

^22^

https://docs.google.com

^23^

https://authorea.com

^24^

https://overleaf.com

^25^

https://doi.org/10.17605/osf.io/yxajs

^26^

http://enigma.ini.usc.edu

^27^

http://brainder.org

^28^

https://fsl.fmrib.ox.ac.uk/fsl/fslwiki/PALM
